# Therapeutic Potential of Polyphenol and Nanoparticles Mediated Delivery in Periodontal Inflammation: A Review of Current Trends and Future Perspectives

**DOI:** 10.3389/fphar.2022.847702

**Published:** 2022-07-12

**Authors:** Putri Ayu Jayusman, Nurrul Shaqinah Nasruddin, Nurul Inaas Mahamad Apandi, Norliwati Ibrahim, Siti Balkis Budin

**Affiliations:** ^1^ Department of Craniofacial Diagnostics and Biosciences, Faculty of Dentistry, Universiti Kebangsaan Malaysia, Kuala Lumpur, Malaysia; ^2^ Centre for Diagnostic, Therapeutic and Investigative Studies, Universiti Kebangsaan Malaysia, Kuala Lumpur, Malaysia

**Keywords:** periodonditis, inflammation, nanotechnolody, nanoencapsulation, polyphenol

## Abstract

Periodontitis is an oral inflammatory process involving the periodontium, which is mainly caused by the invasion of periodontopathogenic microorganisms that results in gingival connective tissue and alveolar bone destruction. Metabolic products of the oral pathogens and the associated host immune and inflammatory responses triggered are responsible for the local tissue destruction. Numerous studies in the past decades have demonstrated that natural polyphenols are capable of modulating the host inflammatory responses by targeting multiple inflammatory components. The proposed mechanism by which polyphenolic compounds exert their great potential is by regulating the immune cell, proinflammatory cytokines synthesis and gene expression. However, due to its low absorption and bioavailability, the beneficial effects of these substances are very limited and it hampers their use as a therapeutic agent. To address these limitations, targeted delivery systems by nanoencapsulation techniques have been explored in recent years. Nanoencapsulation of polyphenolic compounds with different carriers is an efficient and promising approach to boost their bioavailability, increase the efficiency and reduce the degradability of natural polyphenols. In this review, we focus on the effects of different polyphenolic substances in periodontal inflammation and to explore the pharmaceutical significance of polyphenol-loaded nanoparticles in controlling periodontitis, which may be useful for further enhancement of their efficacy as therapeutic agents for periodontal disease.

## Introduction

Periodontitis contributes to a significant public health problem globally due to its high prevalence, economic impact and health consequences (Dom et al., 2012; Marcenes et al., 2013). As a common complex inflammatory disease of the oral cavity, it has been estimated that severe periodontitis affects 11% of the world population in last decades (Kassebaum et al., 2014). Increasing burden of severe periodontitis is suggested to be partly due to an increase in life expectancy in the growing world population. In addition, recent study has reported that half of the adult population worldwide have at least one tooth with apical periodontitis, which is usually asymptomatic and underestimated (Tibúrcio-Machado et al., 2021). Apart from the local effects on the dentition and tooth-supporting tissues, oral diseases may also have an impact on systemic health (Scannapieco and Cantos 2016).

Diseases of the periodontium such as gingivitis and periodontitis are preventable with the application of correct preventive strategies. Nonsurgical treatment modalities remain the gold standard for managing periodontal diseases. Dental cleaning, gingival scaling/root planing (SRP) and proper oral hygiene are among the periodontal treatments available that aim to reduce inflammation, pocket depth and clinical attachment gain (Plessas 2014). In scaling and root planning procedure, antibiotics have been used as adjunct microbial therapy in periodontal pockets through systemic or topical administration (Leszczyńska et al., 2011). According to literature, deep periodontal pockets pose a great challenge for nonsurgical periodontal treatment and in cases where surgical therapy cannot be undertaken, SRP alone may not be sufficient. Bacteria that penetrate the gingival tissue may not be eliminated by mechanical instrumentation hence local and systemic antibiotics are used as an adjunctive modality (Ahad et al., 2016).

The application of non-surgical or surgical periodontal therapy in certain cases as well as antimicrobial therapy however is not wholly successful. In addition to its unwanted side effects, the rise of antimicrobial resistance among patients with severe periodontitis necessitates new strategies in managing periodontal inflammation (Kwon et al., 2021). Based on a recent report in the management of periodontitis, the use of the highest dosage of antibiotic for the shortest duration of time should be considered by clinicians in order to reduce the risk of antibiotic resistance (McGowan et al., 2018). Other major disadvantages that are related to systemic administration are the availability of insufficient concentration of the drug in gingival crevicular fluid and disturbance of intestinal microflora related to the use of antibiotics. Recent studies have also suggested the use of antimicrobial photodynamic therapy (aPDT) as an adjunct to nonsurgical treatment of deep periodontal pockets, however controversial results were reported. Available evidence on aPDT is still scarce due to the low number of controlled studies and high heterogeneity in the study outcome (Salvi et al., 2020; Zhao et al., 2021).

Natural compounds with the capability to modulate host inflammatory response have gained considerable research attention (Palaska et al., 2013). Host modulation therapy (HMT) using anti-inflammatory and antioxidant agents has been investigated and explored as a non-invasive therapeutic approach for periodontitis (Preshaw 2018; Sulijaya et al., 2019). Plant-derived polyphenols have been known to possess antimicrobial, anti-inflammatory, immunomodulatory and antioxidant properties contributing to its benefits in human health. Such characteristics have been reported as the biological mechanisms involve in reducing the initiation and progression of periodontal inflammation. *In vitro* and *in vivo* studies demonstrated that polyphenol possesses an antimicrobial and immunomodulatory potential in treating and preventing periodontitis (Bunte et al., 2019). However, the use of systemic route administration in particular may yield limited results on account of poor pharmacokinetics and pharmacodynamics properties of polyphenol (Hoda et al., 2019). Despite its great potential, polyphenolic compounds have been associated with limited bioavailability mainly due to its low solubility, poor stability in the gastrointestinal tract (GIT) and low intestinal permeability as well as its extremely short plasma half-life (Lu et al., 2016).

Besides continuing the search for new agents that have anti-inflammatory or bone-regeneration properties, the development of slow-release agents is highly desirable to prevent this prevalent and costly disease. The economic burden of periodontitis is highly associated with its prevalence and cost of treatment and in fact is comparable with that of other chronic diseases (Mohd-Dom et al., 2014; Mohd Dom et al., 2016). One of the strategies to overcome the limitations related to polyphenol bioavailability and to enhance its therapeutic applications is by incorporating them into nanoparticles (Conte et al., 2016; Abdul Rahim et al., 2019). The therapeutic potential of polyphenol in managing periodontal inflammation from recently published studies is discussed and the effects of different polyphenol-loaded nanoparticles are highlighted in this present review.

## Host Immune and Inflammatory Responses in Periodontal Inflammation

Gingivitis and periodontitis are the two most common gum diseases that affect periodontal tissues and the supporting structures of a tooth (Hasturk et al., 2012). Gingivitis is most commonly caused by bacterial plaque accumulation on the tooth surface resulting in gum inflammation. In gingivitis, the inflammatory condition is restricted to the soft-tissue area of the gingival epithelium and connective tissue without affecting the deeper compartments of the periodontium. Nonetheless, gingivitis may progress and develop into periodontitis, and therefore it is also considered as the prerequisite for the onset of periodontitis (Murukami et al., 2018). Periodontitis is a chronic multifactorial inflammatory disease characterized by progressive destruction of the periodontal supporting tissue including periodontal ligament and alveolar bone. It is indicated by gingival inflammation, clinical attachment loss, alveolar bone loss that could be assessed by radiography, presence of periodontal pocket and gingival bleeding (Papapanou et al., 2018).

### Activation of Host Immune and Inflammatory Responses

Inflammation is an innate immune response of the body intended to eliminate the initial cause of tissue injury. Inflammatory responses play a major role in our body defence system against pathogens that also involve wound healing. Hence, inflammation is vital for our health and survival. The mouth provides a suitable environment for the growth of microorganisms. Thus, bacteria are present constantly in the mouth and some of these bacteria can trigger inflammatory response and implicated in oral disease. Most of the cases in periodontal disease are originated from bacterial dental plaque or biofilm accumulated on the teeth that induce host periodontal tissues inflammatory response (Hajishengallis et al., 2020). Both chronic gingivitis and periodontitis are chronic lesions that display inflammation and attempts at healing (Hasan and Palmer 2014).

The earliest process of inflammation involves its response against the invading microorganisms. In a susceptible host, invasion of periodontal pathogens particularly a group of specific Gram-negative anaerobic species in subgingival dental biofilm including *Porphyromonas gingivalis, Tannerella forsythia*, and *Treponema denticola* may result in chronic inflammation. These complex bacteria are predominantly found in deep periodontal pockets of patients with periodontitis (Kwon et al., 2021). The major component of the most Gram-negative bacteria present in the oral biofilm particularly lipopolysachharide (LPS) along with other virulence factors cause stimulation of mast cells and the release of vasoactive amines that cause vasodilation of blood vessels. The release of preformed tumour necrosis factor α (TNF-α) causes a subsequent release of inflammatory mediators in the gingival tissue (Hasturk et al., 2012; Yucel-Lindberg & Båge 2013). The chemoattractant proteins (chemokines) generated at this stage results in the initiation of the first line of defence, which are neutrophils that move and migrate to the site of microbial invasion. This process leads to the release of lysosomal enzymes that may also contribute to tissue degradation (Ge et al., 2015). Neutrophil infiltration is then followed by the activation of macrophages. Macrophages activation play a critical role in the elimination of the invaded bacteria, recruitment of other cells to the site of infection, removal of neutrophils excess, production of cytokines and chemokines and activation of lymphocyte-mediated adaptive immunity (Hasturk et al., 2012).

As lymphocytes and macrophages further invade the tissue, collagen content in gingiva is degraded but the bone at the site of lesion may still be intact. At this point, the associated damage is still reversible as it is still possible for gingival tissues to undergo repair and remodelling. In complete resolution with healing, the outcome of the inflammatory process can be restricted or cleared by the role of neutrophils and macrophages. The destructive inflammatory lesion that results in loss of local collagen due to accumulation of polymicrobial biofilm at the gingiva of the teeth is reversible upon resolution of the inflammation (Van Dyke et al., 2020). At this stage, the process of fibrosis and scar tissue formation may limit the infection but failure to clear the infection might establish a chronic inflammatory lesion.

### Progression From Gingivitis to Periodontitis

It is noted that gingivitis is a major risk factor for periodontitis and it may develop into periodontitis in disease-susceptible individuals in which the host response is ineffective and dysregulated (Yucel-Lindberg and Båge 2013; Hajishengallis 2015). The pathogenesis of periodontal disease can be generally categorized into four stages, based on histopathological examination of the development of periodontal inflammation due to plaque accumulation. These stages are called 1) the initial, 2) the early, 3) the established, and 4) the advanced lesions. The advanced lesion which is also known as the destructive phase is clinically recognised as periodontitis where the inflammation extends deeper, with the formation of periodontal pocket, clinical attachment loss, collagen and bone loss (Hasan and Palmer 2014). The “established lesion” can persist for many years and the progression to an “advanced lesion” marks the transition from chronic and successful defence mechanism to destructive immunopathological mechanism or periodontitis. Recently, a new periodontitis classification scheme has been adopted according to the staging and grading system. Classification of periodontitis based on stages is dependent upon the severity of disease at presentation and the complexity of disease management. Meanwhile, classification of periodontitis based on grades reflect biologic features of the disease including analysis of the rate of periodontitis progression, risk for further progression, possibility of poor treatment outcomes and assessment of the risk that the disease or its treatment could affect the systemic health of the patient (Papapanou et al., 2018).

The exact factors that are responsible for the progression of periodontal diseases are unknown but chronic periodontal diseases involve the interaction of several components, which are the bacterial product or the pathogenicity factor, various cell populations and inflammatory mediators (Yucel-Lindberg and Båge 2013). Interaction between the host and microflora with time may result in dysbiosis of microbiome and dysregulation of host inflammation (Hajishengallis 2015). Colonization of Gram-negative bacteria up to 80% in the gingival sulcus during the establishment of periodontal disease form subgingival plaque, leading to periodontal pockets formation and gum recession (Palaska et al., 2013). Among the major pathogens, *A. actinomycetemcomitants* are more commonly detected in high levels in patients with aggressive periodontitis (Teles et al., 2013). The growth of pathogenic microbes within the dental plaque produces substances that could exacerbate inflammation, which may then lead to tissue destruction and even tooth loss. The immune-inflammatory mechanism in periodontal diseases is in part controlled by an individual’s susceptibility and interaction with environmental factors (Lamont et al., 2018).

The destruction of periodontal tissue and bone resorption are contributed by cellular activation, inflammatory mediators including cytokines, chemokines, prostaglandins and proteolytic enzymes particularly matrix metalloproteinases (MMP). The progression of periodontitis involves the release of prominent cytokines such as TNF-α, which is also involved at the early stage of inflammatory cascade, and IL-1 that are produced by the B-cell/plasma cell (Pan et al., 2019). These two cytokines may induce a number of inflammatory mediators such as IL-6, IL-8, MMP and prostaglandin E_2_ (PGE_2_), which is the most prominent prostaglandin implicated in the pathogenesis of periodontitis. IL-1 exerts different biologic effects on different cells and is an important biological mediator of autoimmune and inflammatory diseases. It plays a crucial role in both innate and adaptive immunity. Among first members of IL-1 identified include IL-1α and IL-1β (Dinarello 2018). 11 known members of IL-1 includes molecules with agonist activity such as IL-1α, IL-1β, IL-18, IL-33, IL-36α, IL-36β, and IL-36γ), receptor antagonists of IL-1Ra, IL-36Ra and two anti-inflammatory cytokines which are IL-37, and IL-38 (Dinarello 2018).

Dental biofilm dysbiosis will stimulate the release of IL-1 proinflammatory cytokines including IL-1α, IL-1β, IL-18, IL-36 from the oral junctional epithelium. These proinflammatory signals combined with bacterial products in the periodontal tissues in turn will stimulate both innate and adaptive immunity response. Hence, both complexes will promote release of inflammatory cell mediators into the periodontium (Papathanasiou et al., 2020). Virulence properties of periodontal pathogens such as lipopolysaccharides, stimulates the release of both IL-1α and IL-1β form oral epithelial cells hence resulting in periodontal destruction. Recently, it has been demonstrated that host defense peptides (HDPs) released from the gingival epithelium leading to accumulation of mast cells (MCs) that induces release of more pro-inflammatory cytokines. Therefore, enhancing further breakdown of periodontal tissue (Chompunud et al., 2020). IL-1β upregulates MMPs secretion hence contributing to augmented vasodilation, chemotaxis of inflammatory cells and degradation of collagen. In addition, osteoclastogenesis is also enhanced leading to elevated activity of bone resorption. A study reported that within gingival biopsies of active periodontal disease, there was reduced expression of inflammasome regulators while expression of messenger RNA (mRNA), NLRP3 and IL-1β were amplified (Aral et al., 2020).

Dendritic cells (DCs) release IL-18 that stimulates differentiation of T helper 1 cell (Th1) and T helper 17 cell (Th17) which in turn upregulates the release of IL-17, TNF-α, and IL-1β, hence contributing to further periodontal destruction. Elevated release of IL-1 proinflammatory has been associated with increased receptor activator of nuclear factor-κB ligand (RANKL) release, thus inducing osteoclasts progenitors responsible for alveolar bone resorption in periodontitis. However, IL-1 family members possessing anti-inflammatory properties play a defence role in periodontitis. Here, they will alleviate the magnitude of periodontal inflammation (Papathanasiou et al., 2020). The production of PGE_2_ by the immune cells, fibroblasts and other resident gingival cells is associated with the formation of osteoclast via RANKL upregulation and osteoprotegerin (OPG) inhibition (Belibasakis & Guggenheim 2011).

Since the development of periodontitis is mediated by bacterial-induced inflammation that lead to an excessive host response, the use of conventional mechanical therapy and pharmacological adjuncts is therefore plausible to control the inflammation (Bartold and Van Dyke 2013). Evidence showed that, adjunctive antibiotics and other anti-inflammatory agents can be effective to inhibit or eliminate periodontopathogenic microorganisms and modulate the inflammatory response of the tissue (Da Rocha Júnior et al., 2015). The use of nonsurgical periodontal therapy with or without antimicrobials remains as standard of care that could mechanically removes dental biofilm but targeting only microbes does not equally favourable in all periodontal patients (Preshaw 2018). In addition, the use of adjunctive antibiotics is associated with the unwanted side effects and should only be recommended for progressive disease (Tilakaratne and Soory 2014). Some of the side effects associated with the most commonly used antibiotics in managing periodontal disease include nephritis, gastrointestinal problems, increased risk of allergy, allergic signs on the skin, disturbance in the nervous system and electrolytes imbalance (Heta and Robo 2018).

This is where other agents with similar properties and biological effects with fewer side effects can take its place in improving and managing periodontitis. In fact, One of the promising therapeutic approaches in managing periodontitis particularly in individuals with a higher risk for periodontitis is by modulation of host inflammatory mediators. The term ‘host modulation therapy’ was initially introduced by Golub et al., 30 years ago (Golub et al., 1998). In the last decades, the efficacy of HMT using anti-inflammatory and antioxidant agents in periodontitis was explored. The two major HMT categories accepted include firstly the modulation of the host’s inflammatory response via inhibition or resolution and secondly modulation of host’s pathologic collagenolytic response within the periodontal soft tissue along with alveolar bone (Van Dyke, 2017; Golub et al., 2018). The adjunctive use of host modulatory agents has been postulated to have a positive implication on the progression of periodontal disease particularly in susceptible patients and in individuals whom conventional therapeutic approach is ineffective (Balta et al., 2021). Recently, polyphenols have been documented to cause immunomodulatory effects by downregulating the proinflammatory cytokines, IL-1 and IFN-γ which could be beneficial to be used as adjunct therapeutic approaches in reducing the burden of various inflammatory diseases (Shakoor et al., 2021).

## Role of Polyphenolic Compounds in Managing Periodontal Inflammation

In recent years, polyphenolic compounds are among plant-derived phytochemicals that have gained remarkable attention due to their low toxicity, compared to allopathic drugs in the treatment of inflammatory diseases (Bellik et al., 2013). Polyphenols are secondary reactive metabolites, and they are abundant in plant-derived food, particularly fruits, seed and leaves. Polyphenols represent a wide variety of active compounds that are divided into several major categories based on the number of phenol rings and their structural elements (Belščak-Cvitanović et al., 2018). They are sub-categorized into phenolic acids (hydrobenzoic and hydroxycinnamic acid), flavonoids (flavones, flavonols, flavanols, isoflavones, flavanones, anthocyanins), stilbenes (resveratrol, piceatannol), lignans (sesamol, pinoresinol, sinol, enterodiol), and others including tannins (hydrolysable, non-hydrolysable, and condensed tannins), lignins, xanthones, chromones, anthraquinones (Singla et al., 2019).

Preservation and maintenance of periodontal health is an important component of oral and overall health (Baehni et al., 2010). A substantial number of studies have been done to ascertain the use of polyphenol as an adjunct to managing inflammatory conditions and hence support its role in the prevention and treatment of periodontal disease. Basically, these natural compounds regulate the inflammatory signalling by modifying the expression of several pro-inflammatory genes in addition to their antioxidative potential (Yahfoufi et al., 2018). *In vitro* and *in vivo* studies have demonstrated that the immune modulatory effect of polyphenols is particularly contributed by its potential in modulating immune cells populations, cytokines production and pro-inflammatory genes expression.

Apart from that, a number of studies supported the antimicrobial effect of polyphenols against a variety of pathogens including periodontal pathogens in complex biofilms. Ideally, prevention and treatment of periodontal diseases should also consist of strategies to eliminate or reduce these biofilms. Dietary polyphenols have been reported to have bacteriostatic/bactericidal activity against microbial species such as *P. gingivalis* (Basu et al., 2018). Since periodontal illnesses are inflammatory diseases of bacterial origin, anti-inflammatory and anti-microbial properties of polyphenols may anticipate various biological mechanisms for reducing the initiation and progression of periodontitis. In this review, we presented several polyphenolic compounds that have been reported to have remarkable anti-inflammatory properties particularly in an experimental model of periodontitis. Antibacterial properties of the selected polyphenolic compounds were also described in the current review.

### Quercetin

Quercetin is categorized as a flavonol, one of the six subclasses of flavonoids compounds (Li et al., 2016). The antimicrobial and anti-inflammatory properties of quercetin have been found to be effective to restrict inflammatory reaction in periodontitis. During acute and chronic inflammation in periodontitis, high amount of TNF-α generated by activated macrophages may lead to periodontal tissues degeneration (Noh et al., 2013). A study by Xiong et al. (2019) has demonstrated that all three doses of quercetin (5, 10 and 20 μM) attenuated the production of inflammatory mediators including TNF-α, IL-1β, IL-6, and IL-8 in *Porphyromonas gingivalis* (*P. gingivalis*) LPS-treated human gingival fibroblast. In addition, quercetin has been found to suppress LPS-induced nuclear factor kappa-B (NF-κB) activation in a dose-dependent manner (Xiong et al., 2019). Suppression of these inflammatory mediators and its signalling pathway could restrict the initiation and progression of periodontal disease (Muniz et al., 2015).

In an earlier study, Cheng et al. (2010) investigated the effect of quercetin on experimental periodontitis induced by LPS injection and silk ligation (Cheng et al., 2010). The study has reported that 5 days of oral quercetin treatment at the dose of 75 mg/kg reduced LPS-induced osteoclast formation, ligature-enhanced periodontal inflammation and subsequent alveolar bone loss. Inflammation was induced in the quercetin-administered group but not severe enough to cause alveolar bone loss as evidenced from bone micro-computerized tomography (μ−CT) evaluation. Quercetin decreased the area of inflammatory cell infiltration in connective tissue and narrowed connective tissue attachment. However, the findings also reported a similar attachment loss in both ligation and the ligation-plus-quercetin groups. The results indicated that alveolar bone loss may be prevented in the experimental periodontitis but quercetin was unable to prevent attachment loss, which questioned the beneficial effects of quercetin. The author postulated that the study duration might not be sufficient to observe the effect of quercetin on long-term attachment loss in chronic periodontitis.

In another study, the anti-inflammatory properties of 100 mg/kg quercetin were evaluated using a mouse periodontitis model induced by inoculation of A. *actinomycetemcomitans* (Napimoga et al., 2013). Subcutaneous treatment with quercetin has been found to reduce gingival pro-inflammatory cytokines (IL-1, TNF-α, IL-17), and down-regulate adhesion molecule ICAM-1 and osteoclastogenic cytokine RANKL production in *A. actinomycetemcomitans*-induced alveolar bone loss. Alteration in cell-mediated and humoral levels of adaptive immune defence is thought to be involved in the development of experimental bacterial-immune inflammation in periodontal tissue. The potential of quercetin in reducing bone loss was also demonstrated in a study by Taskan and Gevrek (2020). Osteoblastic activity was increased while osteoclastic activity, apoptosis and inflammation were decreased in ligature-induced periodontitis rats treated with quercetin. A study by Demkovych et al. (2021) was carried out to investigate the effects of quercetin on adaptive immunity in relation to the development of experimental bacterial-immune periodontitis. Intramuscular injection of water-soluble quercetin at a dose of 100 mg/kg for 7 days was found to normalize the cellular adaptive immunity indices and reverse the inflammatory process in the periodontal complex.

It was suggested that the inhibition of periodontal pathogen virulent factor could hamper the progression of periodontitis, prevent and control periodontal inflammation. The effect of quercetin on *P. gingivalis* virulent pathogenicity was demonstrated in a study by He et al. (2020). The study found that quercetin inhibits virulence and physiological properties of *P. gingivalis* as indicated by gingipain, hemolytic and hemagglutination activity. Quercetin also could modulate cell surface hydrophobicity, aggregation, biofilm formation and virulence gene expression. These findings suggested that quercetin might be beneficial in the treatment of periodontitis as it could impair the pathogenicity of *P. gingivalis,* a keystone pathogen for periodontal disease. Table 1 summarized the effects of quercetin in periodontal inflammation, *in vivo* and *in vitro*.

**TABLE 1:** The effects of quercetin in periodontal inflammation, *in vivo* and *in vitro*.

Researcher (year)	Type of study	Experimental method/type of induction	Dose/Delivery of polyphenol treatment	Study outcomes
Xiong et al. (2019)	*In vitro*	*P. gingivalis*-LPS-stimulated human gingival fibroblasts (HGFs)	5, 10 and 20 μM prior to LPS stimulation	• No cytotoxic effects on cell viability of HGF
				• Suppress IL-1*β*, IL-6, IL-8, and TNF-*α*
				• Suppress mRNA levels of IL-1β, IL-6, IL-8, TNF-α, p65, IκBα, TLR4 upregulation and PPAR-γ downregulation
				• Inhibit upregulation of TLR4 expression and the phosphorylation of p65 and IκBα
				• Up-regulate PPAR-γ expression
Cheng et al. (2010)	*In vivo*	Experimental periodontitis - daily LPS injection	Oral, 75 mg/kg, 5 days	• Reduced number of osteoclast
				• Apically located bone crests rebounded, more coronal alveolar crest bone levels, less inflammatory cell-infiltrated connective tissue areas and less connective tissue attachments
Napimoga et al. (2013)	*In vivo*	Experimental periodontitis - Oral inoculation of *A. actinomycetemcomitans*	Subcutaneous, 100 mg/kg, 15 days	• No effect on *A. actinomycetemcomitans* Colony-Forming Units (CFU)
				• Inhibit *A. actinomycetemcomitans*-induced bone loss
				• Inhibit IL-1β, TNF-α, and IL-17 production, ICAM-1 and RANKL expression
Taskan and Gevrek (2020)	*In vivo*	Experimental periodontitis - Silk ligation	75 and 150 mg/kg, 15 days	• Reduce alveolar bone loss
				• Decrease TRAP + osteoclast cells, increased osteoblast cells
				• Decrease iNOS, MMP-8, and caspase-3 levels
				• Increase TIMP-1 expression
Demkovych et al. (2021)	*In vivo*	Inoculation of microorganisms mixture diluted with egg protein with complete adjuvant of Freund	Intramuscular, 100 mg/kg, 7 days	• Increase in the blood of the T-helper cell content (CD4^+^), common mature T-lymphocytes (CD3^+^) CD19^+^ and CD16^+^
				• Reduced level of CD8^+^ and NK-cells content
He et al. (2020)	*In vitro*	*P. gingivalis* culture	50 and 100 μM	• Inhibit gingipains, hemolytic, hemagglutination activities and biofilm formation at sub-MIC concentrations
				• Sparce and thinner biofilm formation
				• Modulate cell surface hydrophobicity and bacterial aggregation
				• Down-regulate the expression of virulence genes

### Resveratrol

Resveratrol (trans-3,4,5-trihydroxystilbene) is a common stilbene found in berries, grape skin and in other plants (Gambini et al., 2015). Resveratrol has attracted substantial attention, as it possesses remarkable biological properties and this could be due to its molecular structure that confers its ability to bind to many biomolecules. Based on its promising anti-inflammatory and antioxidant properties, resveratrol has been studied for its prophylaxis and therapeutic potential in controlling periodontal disease. Resveratrol was shown to have a favourable effect on vascular inflammation induced by periodontal pathogen, *P. gingivalis* as it was able to inhibit NF-κB-dependent cell adhesion molecules in monocyte adhesion to the endothelium (Park et al., 2009). Casati et al. (2013) studied the effect of resveratrol that was administered continually on the progression of experimental periodontitis. Findings of the study showed that bone-loss value in ligated molars and unligated teeth in the control group were higher than in the treatment group that was administered with 10 mg/kg resveratrol for 30 days (19 days before periodontitis induction and 11 days after ligature placement). Resveratrol treated group also showed lower concentration of IL-17 in the gingival tissue. Modulation of IL-17 levels in gingival tissue that presents ligature-induced experimental periodontitis suggests the possible biologic mechanism of resveratrol during periodontal inflammation.

It is known that periodontitis is a multicomponent disorder that affects the supporting structures of the teeth including periodontal ligament and the alveolar bone. Bone loss values were found insignificant between resveratrol, curcumin and the combined groups in a study of continuous curcumin and resveratrol administration against the progression of experimental periodontitis. When compared with the placebo group, the concentration of IL-1 was lower in the combined group as revealed by immune-enzymatic assays. However, resveratrol, curcumin and combined groups showed higher IL-4 levels when compared to placebo group. The reduction in IFN-γ level was only observed in the resveratrol group and the difference in TNF-α levels among groups were not significant. Whether agents were added singly or in combination, both poplyphenolic compounds were able to attenuate alveolar bone loss in the experimental model of periodontitis. Nonetheless, the effects were neither synergistic nor additive (Corrêa et al., 2017). In another study, resveratrol derivative-rich melinjo seed extract was found to have a potent impact on inflammation-induced bone loss in a murine model of established periodontitis as indicated by a reduction in osteoclast differentiation (Ikeda et al., 2018) The production of IL-1β in gingival tissue was reduced but no significant changes in IL-6, TNF-α, and IL-17 levels were observed.

The antibacterial effects of resveratrol against periodontal pathogens *P. gingivalis*, *T. forsythia* and *A. actinomycetemcomitans* were studied by Cirano et al. (2016) in experimental model of periodontitis. The study however found that resveratrol does not exert positive effects on microbiological outcomes suggesting other mechanisms could contribute to its promising effect in controlling periodontitis. Nevertheless, in a recent study, resveratrol has been found to prevent biofilm formation and inhibit the virulence properties of *P. gingivalis* by reducing the expression of virulence factor genes including fimbriae and proteinases (Kugaji et al., 2019). The effect of resveratrol in *in vivo* and *in vitro* model of periodontal inflammation has been summarized in Table 2.

**TABLE 2:** The effects of resveratrol in periodontal inflammation, *in vivo* and *in vitro*.

Researcher (year)	Type of study	Experimental method/type of induction	Dose/Delivery of polyphenol treatment	Study outcomes
Park et al. (2009)	*In vitro*	HMECs incubated with *P. gingivalis* LPS	1 µM or 10 µM	• Inhibit the leukocytes adhesion to endothelial cells and to the aortic endothelium by down-regulation of ICAM-1 and VCAM-1
				• Suppress IκBα phosphorylation and nuclear translocation of the p65 subunit of NF-κB in HMECs
				• Suppress NF-κB expression
Casati et al. (2013)	*In vivo*	Experimental periodontitis—Cotton ligation	Gavage, 10 mg/kg, 30 days	• Lower alveolar bone loss in both ligated and unligated groups
				• Lower concentration of IL-17, no changes in in the IL-1b and IL-4 levels
Ikeda et al. (2018)	*In vivo*	Experimental periodontitis—Silk ligation	Intraperitoneal, 0.004% (w/w)	• Lower alveolar bone loss
				• Lower levels of IL-1β, no changes in f IL-6, TNF-α and IL17 levels
				• Inhibit M-CSF/RANKL mediated osteoclast formation and down-regulate osteoclast activity
Cirano et al. (2016)	*In vivo*	Experimental periodontitis—Cotton ligation	Gavage, 10 mg/kg, 30 days	• No difference in the concentration of periodontal pathogens *A. actinomycetemcomitans, P. gingivalis* and *T. forsythia*
				• No difference in the percentage of sites that were positive for periodontal bacteria after therapy
Kugaji et al. (2019)	*In vitro*	*P. gingivalis* culture	MIC and MBC concentration	• Prevent biofilm formation and reduce the expression of virulence factor genes fimbriae (type II and IV) and proteinases (kgp and rgpA)

### Curcumin

Curcumin, a yellow-coloured compound with low molecular weight is the main polyphenols extracted from the rhizome of turmeric plant, *Curcuma longa* L., (family: *Zingiberaceae*) (Wilken et al., 2011). This natural polyphenol has attracted considerable attention due to its nontoxicity and it has been used to treat various inflammatory diseases since long ago. Curcumin (diferuloylmethane) makes up 2–5% of turmeric and no adverse effects were found when it is given even as high as 8 g/day (Bhatia et al., 2014). Therapeutic potential of curcumin was observed in controlling inflammation and bone resorption in periodontitis. The antioxidant, antimicrobial, anti-inflammatory and analgesic properties of curcumin make them a suitable candidate for the management of periodontal diseases (Forouzanfar et al., 2020).

The possible mechanisms of curcumin involved in the suppression of periodontal disease reported in the existing studies are mainly based on its antibacterial and anti-inflammatory properties. *In vitro* studies have shown that curcumin can inhibit various periodontal pathogens growth such as *A. actinomycetemcomitans, F. nucleatum*, and *P. gingivalis* and the formation of biofilms (Shahzad et al., 2015). Curcumin has been found to have anti-biofilm and high antibacterial activity against *P. gingivalis,* which is considered as the main pathogen and major colonizer in host tissues (Shahzad et al., 2015; Izui et al., 2021; Kumbar et al., 2021). On the other hand, curcumin has low antibacterial activity against *S. mitis*, which is a part of the normal flora in the oral cavity and exert no threat to oral health. This result suggested that curcumin may have selective antimicrobial properties. In gene expression studies done by Kumbar et al. (2021), the virulence of *P. gingivalis* was reduced by curcumin as indicated by a reduce expression of genes coding for major virulence factors including adhesions and proteinases.

The occurrence and development of periodontitis involve the production of a vast number of inflammatory mediators. A recent study carried out by Xiao et al. (2018) showed that the production of IL-β and TNF-α were attenuated in rat gingival fibroblasts supplemented with 10 and 20 μM curcumin. The ratio of OPG/RANKL and the activation of NF-κB induced by LPS *in vitro* were also inhibited. In the same study, an *in vivo* ligation-induced experimental periodontitis showed that curcumin at the dose of 30 and 100 μg/g could alleviate gingival inflammation and modulated collagen fibre and alveolar bone loss as observed in histological and micro-CT results. An earlier study by Zhou et al. (2013) reported that intra-gastric curcumin administration at the dose of 100 mg/kg for 30 days could reduce alveolar bone loss in ligature-induced experimental periodontitis through the suppression of RANKL/RANK/OPG expression and its inflammatory properties (Zhou et al., 2013). Moderate bone resorption and root exposure, and mild bone loss were observed microscopically in curcumin treated animals. The expression of TNF-α and IL-6 in the gingival tissues of experimental rats treated with curcumin was significantly lower than the experimental periodontitis animal. In line with this study, several other studies also reported that curcumin can modulate the inflammatory response, suppress the pro-inflammatory cytokines particularly TNF-α and IL-6 in ligature-induced experimental periodontitis rat model (Guimarães et al., 2011) and LPS-induced periodontitis rat model (Guimaraes et al., 2012). Though curcumin is effective in inhibiting cytokine gene expression at mRNA and protein levels, the inhibition of NF-κB in the gingival tissue was only observed in the lower dose of curcumin (30 mg/kg), whereas p38 MAPK activation was not affected in both doses. Alveolar bone resorption was not prevented by daily dose of intragastric curcumin administration (30 and 50 mg/kg) for 15 days but its potential anti-inflammatory effect suggests their therapeutic potential in periodontal disease. Table 3 summarized the effects of curcumin in periodontal inflammation, *in vivo* and *in vitro*.

**TABLE 3:** The effects of curcumin in periodontal inflammation, *in vivo* and *in vitro*.

Researcher (year)	Type of study	Experimental method/type of induction	Dose/Delivery of polyphenol treatment	Study outcomes
Shahzad et al. (2015)	*In vitro*	Inoculum suspension of *S. mitis* in artificial saliva	Planktonic minimum inhibitory concentration	• Inhibit adhesion of *S. mitis*, and biofilm formation and maturation
Izui et al. (2021)	*In vitro*	*P. gingivalis* outer membrane vesicles (OMV) induced cytotoxicity in HGE cells	0, 5, 10, 20 μg/ml	• Suppression of IL-6, IL-1β, and TNF-α gene expressions
				• Inhibit the cytotoxic effects of OMVs on cellular migration, adherence to and entry of cells, and cellular apoptotic death
Kumbar et al. (2021)	*In vitro*	*P. gingivalis* culture	MIC and MBC concentration (62.5 and 125 µg ml−1)	• Prevent bacterial adhesion and biofilm formation in a dose-dependent manner
				• Reduce the expression of genes coding for major virulence factors (Adhesions—fmA, hagA, and hagB. Proteinases - rgpA, rgpB, and kgp)
Xiao et al. (2018)	*In vitro*	LPS-induced gingival fibroblasts	10 and 20 μM	• Decrease IL-1β and TNF-α production, OPG/sRANKL ratio and NF-κB activation
	*In vivo*	Experimental periodontitis—silk ligation	30 and 100 μg/g	• Reduce alveolar bone loss, gingival inflammation and collagen fiber destruction
Zhou et al. (2013)	*In vivo*	Experimental periodontitis—nylon thread ligation	Oral gavage, 100 mg/kg, 30 days	• Lower bone resorption, RANKL, RANK, OPG, TNF-a and IL-6 expression
Guimarães et al. (2011)	*In vivo*	Experimental periodontitis—cotton ligation	Oral gavage, 30 and 100 mg/kg, 15 days	• No effect on bone resorption
				• Inhibit NF-κB activation but not p38 MAPK
				• Inhibit IL-6 and TNF-a gene expression
Guimarães et al. (2012)	*In vivo*	LPS injection in the gingival tissues	Oral gavage, 30 and 100 mg/kg, 15 days	• Inhibit NF-kB (lower dose), no effect on p38 MAPK
				• Reduce inflammatory infiltrate, increased collagen content and fibroblastic cell numbers

### Proanthocyanidins

Proanthocyanidins are condensed tannins that take the form if oligomers or polymers of monomeric flavan-3-ols produced as an end product of flavonoid biosynthesis pathway (Rauf et al., 2019). The flavan-3-ols are catechin, epicatechin or their substituted derivatives. It is termed as condensed tannins for its capability to form insoluble complexes with carbohydrates and proteins (Balalaie et al., 2018). Proanthocyanidins are highly hydroxylated structures that is categorized according to the number of hydroxyl substitutions in the B ring in which one hydroxyl substitution refers to propelargonidin, two hydroxyl substitution refers to procyanidin and three hydroxyl substitution refers to prodelphinidin. The therapeutic potential of proanthocyanidins emerges from their unique chemical structure (Luca et al., 2020).

The antibacterial activity of proanthocyanidins has been substantially reported in literature (Nawrot-Hadzik et al., 2021). A study by Savickiene et al. (2018) revealed that proanthocyanidins had a unique antibacterial property that could selectively targets the keystone periodontal pathogens viability such as *P. gingivalis* while preserving the beneficial oral commensal *S. salivarius*. Lagha et al. (2018) reported the antibacterial and anti-biofilm effects of proanthocyanidins against *A. actinomycetemcomitans*. The treatment with proanthocyanidins reduced the growth of *A. actinomycetemcomitans* and resulted in a loss of bacterial viability as indicated by the damage to the bacterial cell membrane. Proanthocyanidins also possessed an anti-biofilm activity against *P. aeruginosa* as reported by Ulrey et al. (2014) and its anti-virulence potential was further investigated in a study by Maisuria et al. (2016).

Apart from its ability to inhibit biofilm formation and adhesion of periodontopathogenic bacteria, the therapeutic effect of proanthocyanidins with regards to periodontal disease include its potential to inhibit cytokine production by immune and mucosal cells and its capability to inhibit MMP production (Bonifait and Grenier 2010). Their capability to inhibit MMP and dentin cross-linker activity have been reported as an additional notable benefit of proanthocyanidins (Balalalie et al., 2018). The anti-inflammatory properties of proanthocyanidins were revealed by the attenuation of pro-inflammatory cytokines secretion (IL-1β, TNF-α, IL-6) as well as MMP-3 and MMP-9 secretion by macrophages stimulated with *A. actinomycetemcomitans* (Lagha et al., 2018). In another recent study, Jekabsone et al. (2019) demonstrated a strong antibacterial, anti-inflammatory and gingival tissue protecting properties of proanthocyanidins in periodontitis mimicking condition. Proanthocyanidins fraction has been found to have a stronger efficiency in suppressing caspases as indicated by the level of caspase-3 and caspase-8, and preventing mediator release as indicated by IL-8 and PGE_2_ secretion from gingival fibroblast, and IL-6 secretion from peripheral blood mononuclear cells. In an earlier study, proanthocyanidins-enriched cranberry fraction has been shown to inhibit the production of MMP-3 and MMP-9 in LPS-induced gingival fibroblasts (Bodet et al., 2007). Gingival fibroblasts, the most abundant cells found in periodontal tissues are actively involved in the host inflammatory response to oral pathogens and is known to mediate local tissue destruction in periodontal disease. The productions of IL-6, IL-8 and PGE_2_ by gingival fibroblast stimulated by LPS were inhibited with the treatment of proanthocyanidins-enriched cranberry fraction (Bodet et al., 2007). The effects of proanthocyanidins in *in vivo* and *in vitro* model of periodontal inflammation has been summarized in Table 4.

**TABLE 4:** The effects of proanthocyanidins in periodontal inflammation, *in vivo* and *in vitro*.

Researcher (year)	Type of study	Experimental method/type of induction	Dose/Delivery of polyphenol treatment	Study outcomes
Savickiene et al. (2018)	*In vitro*	*P. gingivalis* and *S. salivarius* culture	0.02–0.09 g/ml	• Strong antioxidant capacity
				• Reduce the viability of both *P. gingivalis* and *S. salivarius*
Lagha et al. (2018)	*In vitro*	*A. actinomycetemcomitans* culture	0–500 μg/ml	• Reduce the growth of *A. actinomycetemcomitans* and prevent biofilm formation
				• Loss of bacterial viability in preformed biofilms
				• Protect the oral keratinocytes barrier integrity from damage and macrophages from the deleterious effect of leukotoxin Ltx-A
				• Inhibit the secretion of IL-1β, IL-6, CXCL8, TNF-α, MMP-3, MMP-9, and sTREM-1 and activation of the NF-κB signaling pathway
Ulrey et al. (2014)	*In vitro*	*P. aeruginosa culture*	0–100 μg/ml	• Reduce *aeruginosa* swarming motility and inhibit biofilm formation
				• Up-regulate 12 proteins related to iron siderophores or cation transporters and proteins involved in amino acid synthesis
				• Down-regulate 2 proteins related to ATP synthesis and several proteins involved in DNA and RNA synthesis
Maisuria et al. (2016)	*In vivo*	*Drosophila melanogaster infected with P. aeruginosa*	200 μg/ml	• Reduce the production of N-acylhomoserine lactone (AHL)-mediated quorum sensing (QS)-regulated virulence determinants by reducing the level of AHLs produced by the bacteria
				• Inhibit the expression of AHL synthases LasI/RhlI and QS transcriptional regulators LasR/RhlR genes
Jekabsone et al. (2019)	*In vitro*	Rat Gingival Fibroblast Cell Culture	100 μg/ml	• Supress Staphylococcus and *Aggregatibacter* compared to *Escherichia* and prevent *A. actinomycetemcomitans* and LPS-induced death of fibroblasts
				• Decrease LPS-induced release of IL-8 and PGE2 from fibroblasts and IL-6 from leukocytes
				• Block IL-1β, iNOS, and surface presentation of CD80 and CD86 expression in LPS + IFNγ-treated macrophages, and IL-1β and COX-2 expression in LPS-treated leukocytes
Bodet et al. (2007)	*In vitro*	LPS-stimulated gingival fibroblasts	0, 10, 25 or 50 μg/ml in non-dialysable material	• Inhibit IL-6, IL-8, and PGE2 responses of gingival fibroblasts
				• Inhibit fibroblast intracellular signaling proteins, reduce cyclooxygenase 2 expression

## Nanoparticles as Potential Delivery System of Polyphenol

Despite promising biological activities of natural polyphenol, several limitations have been addressed in relation to their bioavailability. The absorption of polyphenolic compounds is negatively affected by its molecular size and their pharmacokinetics is modified by pre-systemic metabolism and gastric environment, which is highly acidic (Neves et al., 2012). Polyphenols were metabolized extensively during their transport across the small intestine and liver causing remarkable alteration of the redox potential. Consequently, the small proportion of polyphenolic compounds that are available following oral administration limits the activity and beneficial health effects of polyphenols. For instance, only a trace amount of curcumin available in blood plasma even after high dose intake, and after rapid metabolization of orally administered curcumin, they form several reduced products in the intestine and is most excreted in the urine and faeces (Niu et al., 2012). Low solubility and stability, short biological half-life and rapid elimination of polyphenolic compounds hinder their clinical application and thus lead the way towards the establishment of systems that could deliver these substances effectively.

The potency of many species of medicinal plants is determined by the availability of the active compounds. Recent research has proposed to combine herbal medicine with nanotechnology because nanoencapsulated particles are expected to potentiate the action of the plant extracts, reduce the required dose and undesirable effects as well as improving its activity (Bonifácio et al., 2014). Nanomedicine is an on-going research area that applies nanotechnology to medical intervention for prevention, diagnosis and treatment of diseases (Chang et al., 2015). Nanotechnology involved the production, processing, and application of particles with diameter ranging from 1 to 1,000 nm (Etheridge et al., 2013). Encapsulation is a process that incorporates an active compound or substances within a carrier material or another substance (Nedovic et al., 2011). The goal of encapsulation is to reduce the damage of sensitive and labile bioactive agents and to protect them from unwanted circumstances (Jafari 2017). Nanoencapsulation is then can be defined as a process of encapsulating a substance within another material at sizes on the nano-scale.

The incorporation with nanoparticles would basically protect the substances against chemical and enzymatic degradation (Muhamad et al., 2014). Nanoparticles have been used in advanced drug delivery systems as the size and surface characteristics of nanoparticles can be manipulated easily which prompt the use for passive and active drug targeting. In addition, nanoscale drug delivery systems could also enhance the solubility of hydrophobic compounds in aqueous medium. Since the biodistribution and clearance of substances from the body can be altered, the therapeutic efficacy can be increased and reduction in side effects can be achieved by the use of nanoparticles as carrier (Liang et al., 2017). Collectively, the nanoencapsulation process aims to enhance the properties of active compounds and to transport them to the target destinations more effectively (Esfanjani and Jafari 2016). Basically, the drug is made active in the targeted area at pre-determined release rate over a period of time (Harini & Kaarthikeyan 2014).

The process of encapsulating one compound to another involves several methods according to their chemical, physical and physiochemical properties (Conte et al., 2016). Chemical nanoencapsulation refers to polymerization of monomers through the addition of a cross-linker in the external phase while physical nanoencapsulation involves the interaction of the vector material with the encapsulated molecules when both are aerosolized or atomized (Wais et al., 2016). Physiochemical processes involve the formation of stable nanometre size drug nano-suspensions or nanoparticles through the reduction of particle size (Merisko-Liversidge & Liversidge 2011). Nanocarriers for phenolic compounds can be roughly divided into polysaccharide- and protein-based delivery systems (Milinčić et al., 2019). Substances such as cyclodectrins, polymeric nanoparticles, nanomicelles, food-protein nanoparticles, zein nanoparticles, gelatin nanoparticles and films, chitosan, lipid nanocarriers, or protein-polysaccharide complex nanoparticles are suitable to be used as carriers for nanoencapsulation of polyphenolic compounds. The interactions between polyphenol and nanocarrier can improve its bioavailability, prevent extensive degradation in the GIT, enhance its delivery to the targeted sites, or even provide stability during the storage or processing. Hence, the application of polyphenol-loaded nanoparticles is an interesting means to improve their overall activity.

## Nanoparticles Mediated Delivery in Managing Periodontal Inflammation

Nanoencapsulation of polyphenolic compounds could overcome the drawbacks related to its instability, limited bioavailability and short half-life *in vivo* and *in vitro* (Fang and Bhandari 2010). The most frequently encapsulated polyphenols reported are quercetin, catechins, epigallocatechin, epigallocatechin-gallate (ECGC), curcumin, eugenol and tea polyphenols (Milinčić et al., (2019). The incorporation of these polyphenols with nanoparticles could be one of the promising approaches to enhance their efficacy as therapeutic agents for managing periodontal diseases.

Rutin is a glycoside that comprise of flavonolic aglycone quercetin along disaccharide rutinose (Ganeshpurkar and Saluja 2017). Rutin or rutin glycoside of quercetin has been reported to have a number of pharmacological activities including anti-inflammatory and antioxidant properties (Wang et al., 2019). The mechanism in which it inhibits oxidative stress and inflammatory reactions in animal models is through the regulation of MAPK pathway (Ganeshpurkar and Saluja 2017). Xu et al. (2020) evaluated the therapeutic effect of local rutin application on gingiva of periodontitis rats. In the study, rutin was incorporated with poly-lactic-co-glycolic acid (PLGA) nanoparticles by chemical precipitation method to improve its bioavailability and to make it more targeted. PLGA is among the widely used biodegradable organic polymer as it has a good biocompatibility, non-toxic and has passed FDA certification. Findings of the study showed that local application of rutin-loaded PLGA nanospheres inhibit the inflammatory reaction in LPS-induced periodontitis that may be due to downstream target effect of rutin combined with prostaglandin endoperoxide synthase 2 and downregulation of NFκBIα.

In a recent study by Wang et al. (2019), the incorporation of quercetin onto nano-octahedral ceria by chemical bonding has been found to be efficient in reprogramming pro-inflammatory macrophages to the anti-inflammatory phenotype that eventually could alleviate inflammation. Post subgingival injection of quercetin-loaded nanoceria was also found efficient to decrease local periodontal inflammation in a rat model of periodontitis induced by *P. gingivalis* injection. This study discovered that quercetin and cerium oxide (CeO_2_) nanoparticles (nanoceria) have synergistic and intense regulation on host immunity against periodontal disease. It is well documented that macrophage serves as the first line of host immune defense against periodontal pathogen infection as it is involved during the onset and resolution of inflammation (Yu et al., 2016). This nanocomposite was able to modulate the phenotypic switch of macrophages by inhibition of M1 (pro-inflammatory) polarization and also promotion of M2 (anti-inflammatory) polarization. Previously, quercetin has been proven to be able to modulate macrophage that eventually gives rise to efficient anti-inflammatory activity (Li et al., 2016; Hu et al., 2019). In the recent study, CeO_2_ nanoparticles were shown to inhibit the polarization of M1 macrophage by suppressing the inflammatory cytokines expression and arresting NF-κB signal pathway (Selvaraj et al., 2015). It has been reported that quercetin could also drive M2 phenotype macrophage polarization (Tan et al., 2020) and this is crucial as M2 macrophage activation could down-regulate gingival inflammation, prevent alveolar bone loss and more interestingly promote periodontal tissue regeneration. Incorporation of quercetin with cerium oxide nanoparticle may exhibit great potential in treating periodontitis.

Though resveratrol is well tolerated by humans, it is rapidly metabolized, leading to a short half-life and insubstantial effectiveness (Cottart et al., 2010). Researchers have mainly focused on increasing the absorption of resveratrol by increasing the residence time and lengthening its activity by incorporating it in biopolymers and lipids (Augustin et al., 2013; Sessa et al., 2014). A study by Giménez-Siurana et al. (2020) evaluated the therapeutic potential of silk fibroin nanoparticles loaded with resveratrol in diabetic-induced periodontitis. The link between periodontitis and other systemic diseases such as diabetes has been widely reported. Proinflammatory factors specifically IL-6, IL-1β and TGF-1β are implicated in both diseases. In the recent study, the levels of IL-1β and IL-6 were significantly decreased with the administration of resveratrol-loaded silk fibroin nanoparticles in the experimental animal. The reduction of these two significant proinflammatory cytokines indicates the recovery from periodontitis (Corrêa et al., 2017). This effect is contributed not solely by silk fibroin nanoparticles but also due to the anti-inflammatory activity exhibited by polyphenol resveratrol that has undergone an encapsulation process (Giménez-Siurana et al., 2020).

The therapeutic potential of curcumin on pathologic bone resorption *in vivo* may be dependent on the dose, route of administration and the type of experimental model used (De Almeida Brandão et al., 2019). The heterogeneity of findings associated with the use of natural curcumin may also be contributed by other variables such as the source, type of vehicle and the pharmacokinetic-related issues including their short half-life and low absorption rate in the GIT. Different encapsulation techniques such as curcumin-based nanoparticles formula and curcumin structure modification are among the latest approaches that can be applied to increase the bioavailability of curcumin *in vivo* but studies are still scarce. The use of alternative vehicles such as lipid-, chitosan- or hydrolysed corn protein associated nanoparticles formulation can be used to improve the pharmacodynamics and biological properties of curcumin (Shome et al., 2016; Wang et al., 2016). A study by Zambrono et al. (2018) reported the effect of curcumin-loaded nanoparticle in LPS-induced model of experimental periodontal disease but instead of using systemic route, this study investigates the local administration of polylactic acid and co-glycolic acid nanoencapsulated curcumin. These locally administered nanoparticles showed 15 times increase of curcumin half-life in the plasma of rats (Khalil et al., 2013). Apart from that, the application of nanoparticles also enables chemical modification that could specify its absorption in the given tissue or cell type and modify their absorption process to avoid macropinocytosis and liposome degradation or allow the tracking of its sub-cellular localization by the covalent binding with fluorescent molecules (Paka and Ramassamy 2017). Local application of nanocurcumin by direct injection into the gingival tissues twice a week in the LPS-induced model was shown to inhibit inflammatory bone resorption indicated by micro-CT analysis (Zambrano et al., 2018). This finding is explained by the reduction of osteoclast numbers, neutrophils (PMNs) and mononuclear cells numbers as indicated by histomorphometric analysis. The reduction of inflammatory cells infiltration suggests the anti-inflammatory effect of local nanocurcumin administration and this result is further supported by the attenuation of both signalling pathways in the gingival tissues, the p38 MAPK and NF-κB. In the earlier studies, systemic administration of curcumin in lipid vehicles was found to inhibit NF-κB activation but not p38 MAPK in LPS-induced experimental periodontitis (Guimarães et al., 2011; Guimaraes et al., 2012).

**FIGURE 1:**
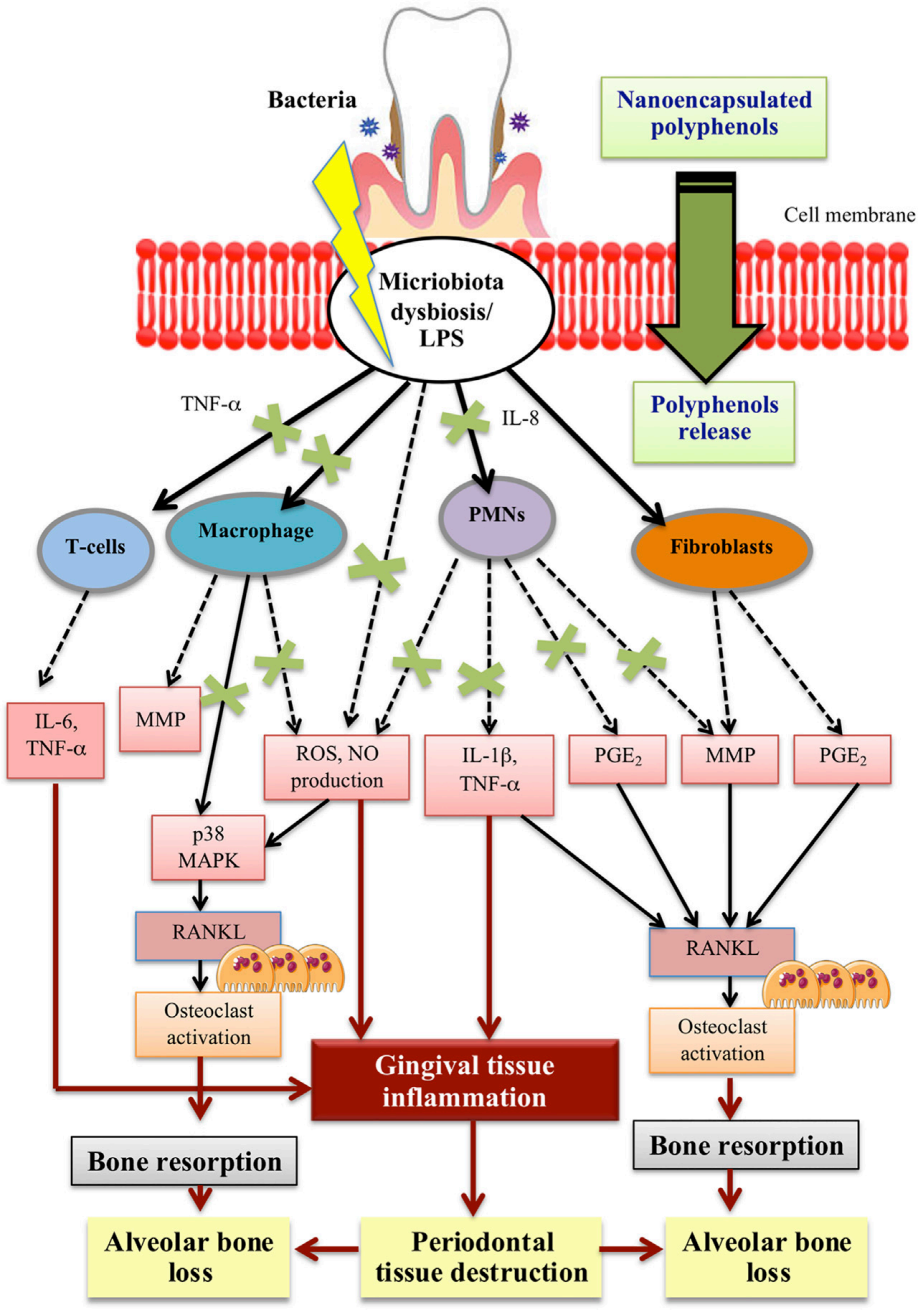
Diagrammatic representation of the nanoencapsulated polyphenols effect on the main cellular pathways involve in periodontal inflammation.

Tea is very rich in polyphenolic compounds mainly flavonoids including epicatechin (EC), epigallocatechin, epicatechin-3-gallate (ECG) and apigallocatechin-3-gallate (EGCG) (Kanwar et al., 2012). In dentistry, the use of catechin for the treatment of dental caries, periodontal and pulp diseases have been documented (Azmi et al., 2020). However, like other polyphenolic compounds, issues on its poor stability and low operational bioactivities lead to the unsatisfactory effect of tea polyphenol. Since previous methods of encapsulating tea polyphenol require tedious procedure, Tian et al. (2021) have developed a one-step polyphenolic condensation reaction that functionalized EGCG, the green tea derivative nanoparticles. The potent antioxidant capacity of EGCG-based nanoparticles was found to improve the chemical stability of epigallocatechin gallate. In addition, EGCG-based nanoparticles also provide more effective ROS scavenging activity and the expression of pro-inflammatory cytokines is down-regulated by reprogramming macrophages from pro-inflammatory M1 to anti-inflammatory M2 phenotype (Tian et al., 2021). *In vivo* findings showed that subgingival injection of EGCG nanoparticles could inhibit the alveolar bone loss and reduce osteoclastic activity in ligature-induced chronic periodontitis model in rats. EGCG nanoparticles have been found to remove ∼50% ROS *in vivo* more efficiently and safely. Down-regulation of the inflammatory cytokines and stimulation of macrophages differentiation to anti-inflammatory phenotype eventually prevent alveolar bone loss. In light of these findings, the development of ECGC-based nanomaterial provides better biocompatibility and anticipates an effective antioxidant defence mechanism for the treatment of chronic periodontitis. Table 5 summarizes recent studies on the effects of nanopolyphenols/nanoencapsulated polyphenols in the application of periodontal inflammation. Figure 1 illustrate the potential effect of polyphenol nanoencapsulation on the main cellular pathways involved in periodontal inflammation.

**TABLE 5:** *In vivo* and *in vitro* effects of nanopolyphenols/nanoencapsulated polyphenols.

Researcher (year)	Type of study	Type of polyphenols	Experimental method/type of induction	Dose/Delivery of polyphenol treatment	Study outcomes
Xu et al. (2020)	*In vivo*, Sprague-dawley rats	Rutin-loaded PLGA nanospheres	Experimental periodontitis—LPS injection on the gingiva	100 μL of 200 mg/ml Rutin added in 1 ml PLGA nanoparticles	• Decrease inflammatory response, expression of PTGS2 and NFBI⍺
Wang et al. (2019)	*In vitro*	Quercetin-Loaded Ceria Nanocomposite	*P. gingivalis* LPS stimulated RAW 264.7	50 μg/ml	• Decrease M1-related biomarkers (TNF-α, IL-6, and IL-1β)
					• Decrease in p65-positive cell counts and TNF-α-positive cell counts, ratio of IL-1β positive cells
					• Up-regulate all M2 biomarkers and inhibit inflammatory-related CD86 expression
	*In vivo*, Wistar rats	Quercetin-Loaded Ceria Nanocomposite	Experimental periodontitis—*P. gingivalis* injection	Local (subgingival injection), 50 μg/ml, 4 days	• Low relative fluorescence intensity at inflammatory sites
					• Lower number of inflammatory cells. Reduce collagen fibers degradation (high fraction of collagen volume)
					• Lower IL-1β positive cells but high amount of Arg-1 positive cells
Giménez-Siurana et al. (2020)	*In vivo*, Sprague-dawley rats	Silk fibroin nanoparticles loaded with resveratrol	Experimental periodontitis—silk ligation in diabetic rats	Oral gavage, 3 mg/ml, 4 weeks	• Reduce chemical inflammation mediator (IL-6 and TGF-β1)
					• Lower inflammation area, collagen compaction and vessel formation (angiogenesis), smaller thickness of the epithelium of the gum
Zambrono et al. (2018)	*In vivo*, Holtzman rats	Curcumin nanoparticles	Experimental periodontitis—LPS injection on the gingiva	Local (gingival tissue injection), 3 μL, 2x per week	• Inhibit inflammatory bone resorption
					• Decrease osteoclast counts and inflammatory infiltrate
					• Attenuate p38 MAPK and NF-kB activation
Tian et al. (2021)	*In vitro*	EGCG green tea derivative nanoparticles	Raw264.7 cells	20 and 40 μg/ml	• Down-regulate the expression of iNOS, IL-1β, IL-6 and TNF-α. Inhibition in mRNA expression of IL-6, TNF-α and iNOS markers than free EGCG group
					• Increase proportion of cells expressed CD206 (M2 phenotype specific markers) and reduce proportion of cells expressed CD80 (M1 phenotype specific marker)
	*In vivo*	EGCG green tea derivative nanoparticles	Experimental periodontitis—wire ligature	Local (subgingival injection), 50, 200 and 500 μg/ml, every 2 days for 3 weeks	• Reduce the cementoenamel junctions -alveolar bone crest (CEJ-ABC) distance and alveolar bone loss at both day 7 and 21, inhibit progression of bone resorption and alveolar bone loss
					• Reduce the expression of the IL-1β, IL-6 and TNF-α on day 7 and 21
					• Lower total number of inflammatory cells and TRAP-positive osteoclast number. Lower number of osteoclast than in free EGCG group

## Conclusion

In the recent decades, phytochemicals, particularly polyphenols have been reported to have a remarkable therapeutic effect in preventing and/or treating inflammatory diseases. From the preclinical studies, polyphenol showed potential to modulate host immune and inflammatory profile in periodontal disease. However, poor water solubility, stability and bioavailability render the biological effects of polyphenol and limit their future clinical application. These drawbacks can be tackled by the application of nanosize delivery systems, which could increase the solubility and stability of phytochemicals and eventually improve their absorption. In addition, this delivery system could protect the substances from untimely enzymatic degradation or metabolism in the body and hence lengthen their circulation time. This review on the application of polyphenol-loaded nanoparticles may be useful for the enhancement of phytochemical efficacy as therapeutic agents in managing periodontal disease. A number of recent studies have investigated the pharmaceutical significance and therapeutic applicability of nanoparticles advance delivery system in improving and enhancing *in vitro* and *in vivo* performance of polyphenolic compounds, but much has yet to be explored. Comparative studies on advance delivery systems for the delivery of polyphenolic compounds and studies on safety/toxicity profile of polyphenol-loaded nanoparticles are warranted to bring the anti-inflammatory phytochemicals closer to the clinical applications.
